# First Confirmed Case of Leadless Pacemaker Insertion via Bidirectional Glenn Shunt in Ebstein's Anomaly: A Novel Approach to Pacing Challenges

**DOI:** 10.1111/jce.70165

**Published:** 2025-10-28

**Authors:** Robert S. Doyle, Hugo C. Temperley, Cian Murray, Kevin Walsh

**Affiliations:** ^1^ Department of Cardiology, Mater University Hospital Eccles St. Dublin Ireland; ^2^ Department of Radiology, St. James Hospital James's St. Dublin Saint James Ireland; ^3^ Department of Cardiology, St. James Hospital James's St. Dublin Saint James Ireland

**Keywords:** Abbott AVEIR VR, cardiac imaging, chest X‐ray, congenital heart disease, Ebstein's anomaly, Glenn shunt, lead dislodgment, leadless pacemaker, pacemaker complications, transvenous pacing

## Abstract

**Background:**

Ebstein's anomaly presents unique pacing challenges due to complex anatomy and surgical history, often leading to transvenous lead complications.

**Case Presentation:**

A 28‐year‐old male with Ebstein's anomaly, prior Danielson tricuspid valve repair, and bidirectional Glenn shunt presented with acute ventricular pacing loss from dislodgment of a 4 French SelectSecure Medtronic 3830 lead into the left upper lobe pulmonary artery. An urgent procedure involved inserting an Abbott AVEIR VR leadless pacemaker via the Glenn shunt to the right ventricular apex, achieving stable thresholds (pacing threshold 0.5 V at 0.4 ms, R‐wave 8 mV). Multimodal imaging (radiographs, fluoroscopy, angiography, echocardiography) confirmed device position and preserved right ventricular function.

**Conclusions:**

This first confirmed case demonstrates the feasibility and safety of leadless pacemaker insertion via Glenn shunt in complex congenital heart disease, providing a durable alternative to transvenous systems prone to dislodgment.

## 1 Case Report

A 28‐year‐old male with Ebstein's anomaly presented with acute loss of ventricular pacing, identified during a routine pacemaker check. His medical history was complex, including a Danielson tricuspid valve repair, atrial septal defect closure, and a bidirectional Glenn shunt as part of a 1.5 ventricle repair in 2005, followed by extracorporeal membrane oxygenation (ECMO) support and mechanical tricuspid valve replacement later that year due to severe right heart failure. Initial pacing was achieved with an epicardial dual‐chamber pacemaker for complete heart block, complicated by ventricular lead fracture, necessitating a VVI pacemaker with a 4 French SelectSecure Medtronic 3830 lead implanted retrogradely via the Glenn shunt into the right ventricle (RV) in 2017 at age 20. Pacemaker generator changes occurred in 2015 and 2024, with a Medtronic device in situ. Medications included Warfarin (stable international normalized ratios [INRs] 2.5–3.5), Enalapril (10 mg daily), Furosemide (40 mg daily), and Spironolactone (25 mg daily) for heart failure management.

Physical examination revealed a pulse of 62 beats per minute, blood pressure of 128/66 mmHg, oxygen saturation of 98% on room air, and a metallic click with a grade 3/6 pansystolic murmur over the pulmonary area, consistent with the mechanical tricuspid valve. Transthoracic echocardiography, performed annually, confirmed good prosthetic valve function, no paravalvular leak, an RV ejection fraction of 50%–55%, and no evidence of pulmonary hypertension. Despite recent weight gain to 104.7 kg (BMI 30.2 kg/m²), the patient remained asymptomatic, engaging in 5–8 h of weekly exercise (cycling and swimming), though his routine was disrupted by college commitments. Electrocardiography showed pacing spikes without ventricular capture, prompting urgent intervention.

The procedure was performed under local anesthesia with Midazolam (2 mg IV) and Fentanyl (100 mcg IV) sedation. The right internal jugular vein (RIJV) was accessed using ultrasound guidance, with two Proglide pre‐closure sutures placed to ensure hemostasis. A 0.35 inch exchange Safari small curve was initially guided into the right ventricle. To navigate significant perivascular scarring around the RIJV and SVC from prior central venous accesses (2017 lead implantation, 2005 ECMO cannulation), predilation with a 26 F Gore Dryseal sheath was required, chosen for its superior lubricity and shapability compared to standard Abbott AVEIR sheaths, allowing for a smoother passage through tortuous anatomy. A 25‐French Abbott AVEIR introducer sheath was then advanced through the superior vena cava and Glenn anastomosis, and into the right ventricle, traversing multiple acute angles at the superior vena cava‐Glenn anastomosis junction and the tricuspid valve, aided by careful predilation. Once positioned in the RV, the AVEIR introducer sheath demonstrated adequate maneuverability despite its large caliber, allowing relatively straightforward torque and deflection to sample multiple septal sites for optimal pacemaker positioning with minimal resistance. Angiography confirmed good RV function (ejection fraction ~50%) and identified the dislodged SelectSecure lead in the left upper lobe pulmonary artery, explaining the loss of pacing. The Abbott AVEIR VR leadless pacemaker was delivered via the Glenn shunt to the RV apex, through the 25 F introducer sheath achieving a pacing threshold of 0.5 V at 0.4 ms and an R‐wave amplitude of 8 mV, indicating excellent myocardial capture. The RV apex was intentionally selected due to extensive surgical scarring from prior epicardial pacing and tricuspid valve interventions that distorted the RV inflow tract, tricuspid annulus, and basal septum, precluding a standard septal approach. The scarred apical region provided a more accessible and stable target with lower perforation risk owing to established fibrosis, which is more resistant to tearing during helix deployment. The device was released after tether mode showed positional and electrical stability. Warfarin was restarted immediately postprocedure with a heparin bridge until INR reached 2.5. The procedure duration was ~90 min, with no complications. No injury to the pulmonary valve was observed intra‐operatively, with the sheath trajectory via the Glenn shunt and RV apex avoiding the outflow tract. Post‐procedural echocardiography at 24 h and 1 month confirmed stable mild pulmonary regurgitation without new dysfunction.

Follow‐up at 1, 3, and 6 months included pacemaker interrogation (stable thresholds), echocardiography (unchanged RV function and valve performance), and chest radiographs, confirming the leadless pacemaker′s position in the RV apex and the dislodged lead's stable position in the pulmonary artery, with no evidence of migration or thrombus formation.

## 2 Imaging Findings

Imaging played a pivotal role in diagnosis and procedural guidance. Serial chest X‐rays demonstrated the sequence of lead dislodgment: a posteroanterior chest X‐ray initially showed the SelectSecure lead correctly positioned via the Glenn shunt into the RV; subsequent posteroanterior and lateral X‐rays revealed lead retraction into the left upper lobe pulmonary artery, with the Abbott AVEIR VR leadless pacemaker visible in the RV apex. In this case, angiography captured the procedural delivery of the 25‐French sheath through the Glenn shunt, navigating the superior vena cava and Glenn anastomosis to position the leadless pacemaker in the RV apex, with the dislodged lead visible in the pulmonary artery. Fluoroscopy confirmed the final positioning of the leadless pacemaker in the RV apex, with clear visualisation of the device and the retracted lead. Echocardiography corroborated good prosthetic tricuspid valve function (mean gradient 4 mmHg), no paravalvular leak, and preserved RV systolic function, supporting hemodynamic stability postprocedure.

**Figure 1:**
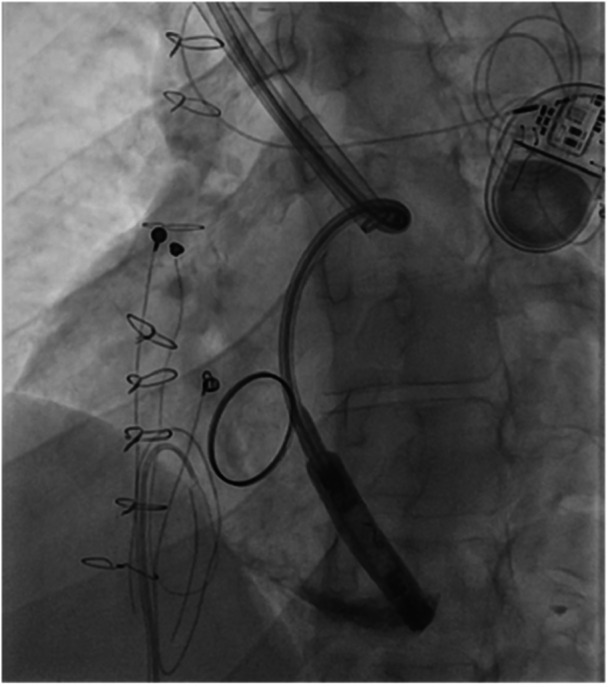
Fluoroscopic image showing initial access via the bidirectional Glenn shunt during leadless pacemaker insertion, with guidewire advancement toward the right ventricle.

**Figure 2:**
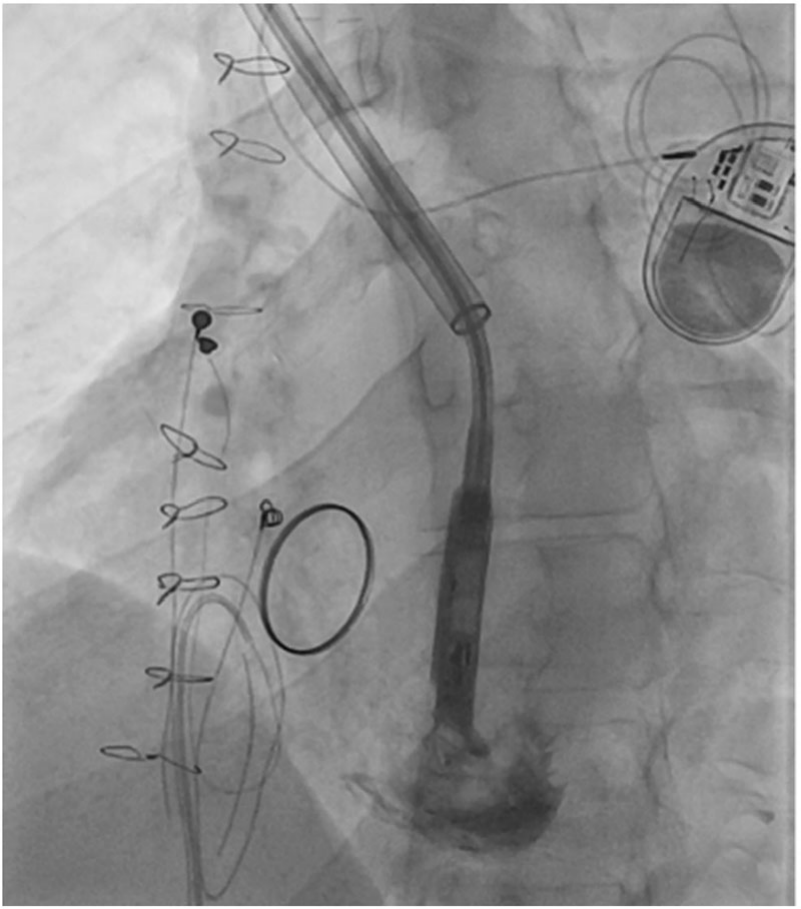
Fluoroscopic view demonstrating catheter navigation through scarred venous anatomy in the Glenn shunt, highlighting challenges due to prior surgical interventions.

**Figure 3:**
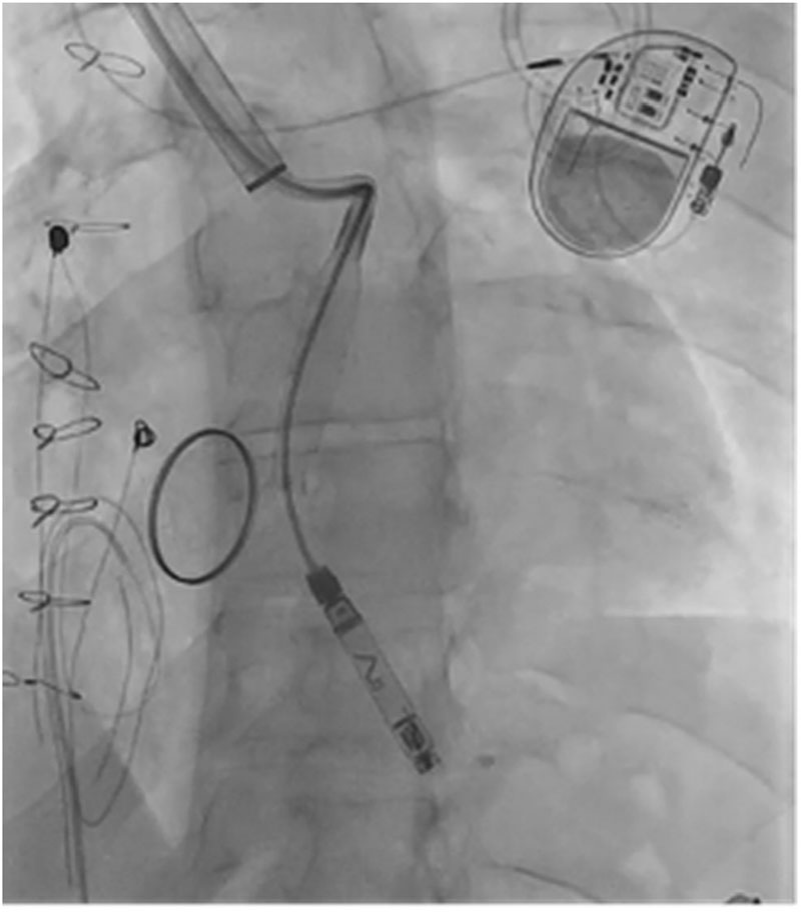
Angiographic confirmation of catheter positioning within the superior vena cava‐pulmonary artery anastomosis, before right ventricular apex targeting.

**Figure 4:**
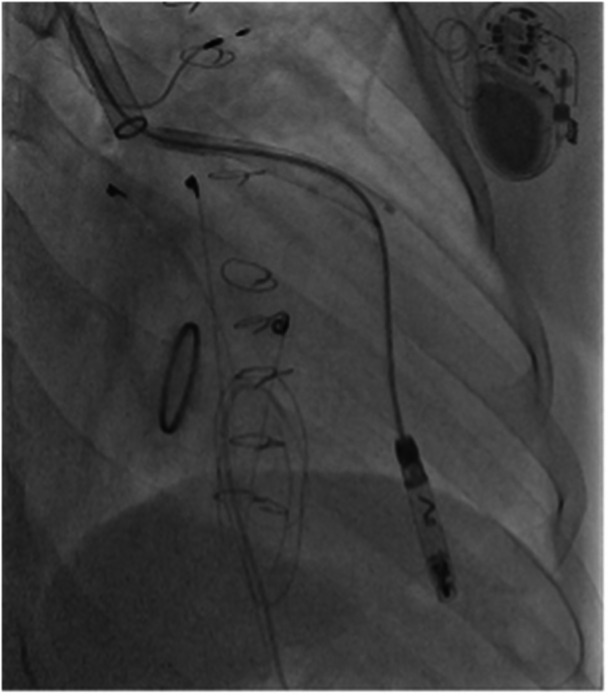
Fluoroscopic image capturing the deployment of the Abbott AVEIR VR leadless pacemaker delivery system into the right ventricular apex.

**Figure 5:**
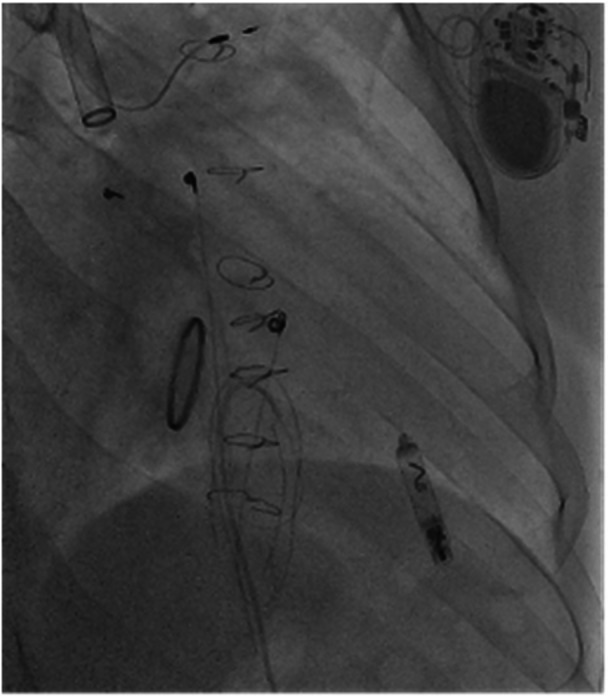
Final fluoroscopic confirmation of the Abbott AVEIR VR leadless pacemaker deployment and fixation in the right ventricular apex, with stable positioning achieved.

Figures 1, 2, 3, 4, 5 demonstrate insertion of the leadless pacemaker in chronological sequence, guided by fluoroscopy.

**Figure 6:**
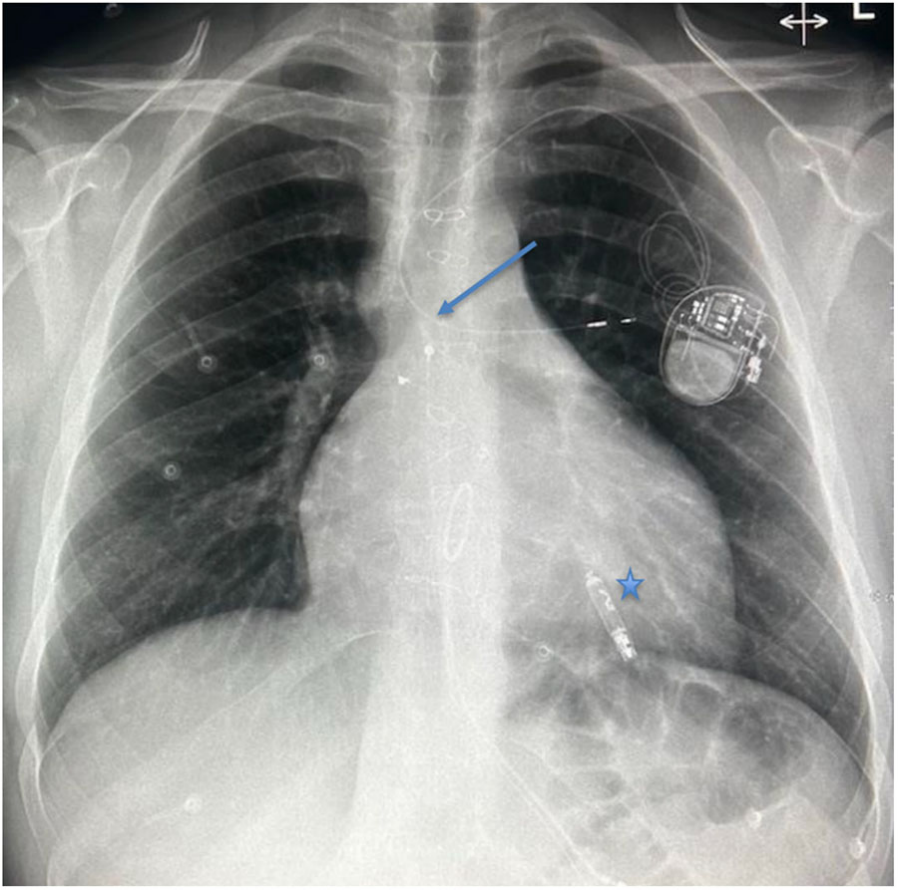
Posteroanterior chest radiograph illustrating the final position of the Abbott AVEIR VR leadless pacemaker in the right ventricular apex, alongside the dislodged SelectSecure Medtronic 3830 lead in a branch of the left pulmonary artery.

**Figure 7:**
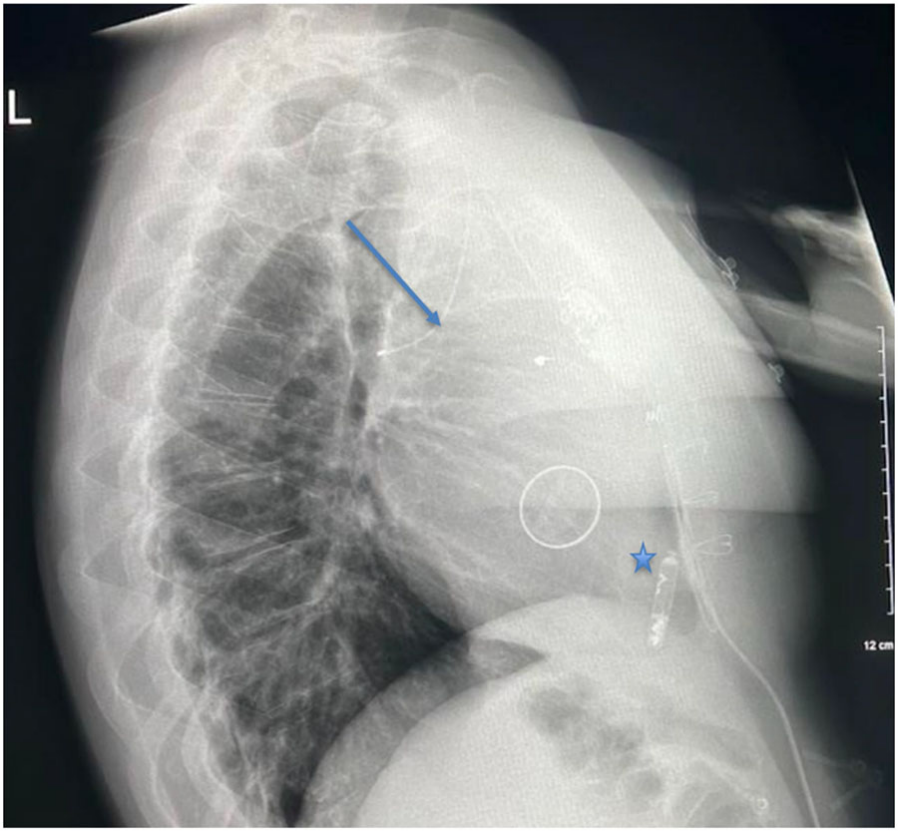
Lateral chest radiograph demonstrating the Abbott AVEIR VR leadless pacemaker in the right ventricular apex and the dislodged SelectSecure Medtronic 3830 lead in the left pulmonary artery branch, confirming post‐procedural stability.

Figures 6, 7 are chest radiographs (posteroanterior and lateral respectively) demonstrating the final position of the Abbott AVEIR VR leadless pacemaker in the RV apex (star), and the dislodged SelectSecure Medtronic 3830 lead in a branch of the left pulmonary artery (arrow).

**Figure 8:**
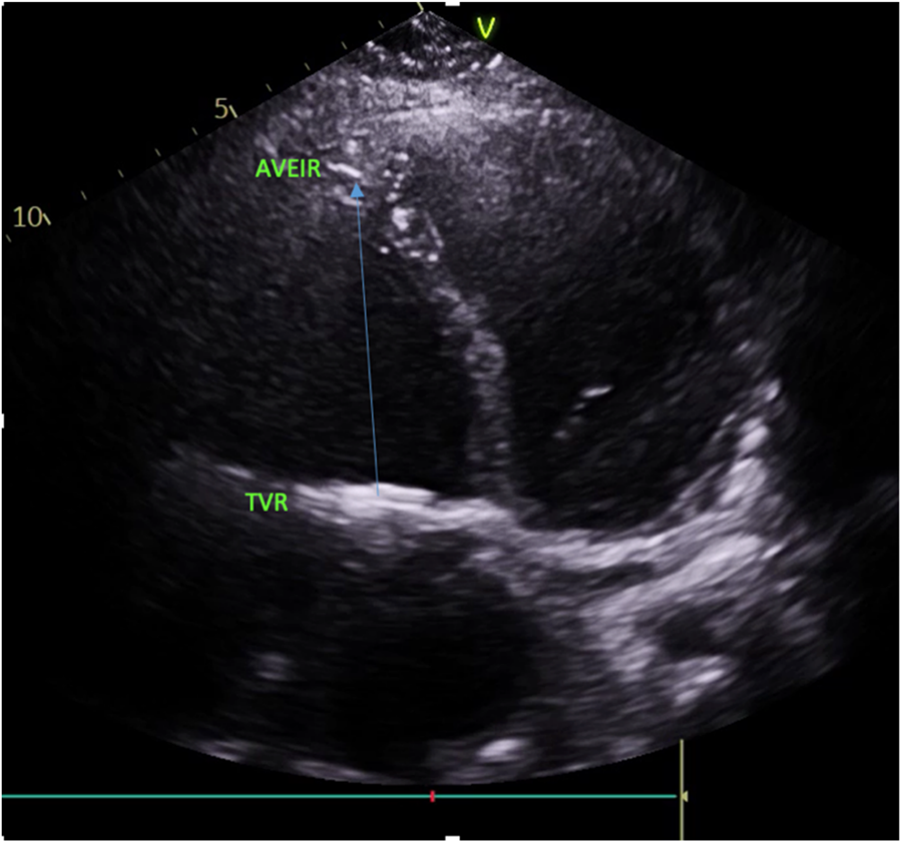
Apical four‐chamber echocardiographic view showing the Abbott AVEIR VR leadless pacemaker in the right ventricular apex, with clear visualization of its distance from the metallic tricuspid valve replacement (TVR) and preserved right ventricular function.

Figure 8 is an echocardiographic image in apical four chamber view demonstrating the Abbott AVEIR VR leadless pacemaker in the RV apex, and its distance from the metallic tricuspid valve replacement (TVR).

## 3 Discussion

This case represents the first confirmed report of a leadless pacemaker (Abbott AVEIR VR) inserted via a bidirectional Glenn shunt in a patient with Ebstein's anomaly with mechanical tricuspid valve replacement, addressing a critical pacing requirement in the complex congenital heart disease (CHD) setting. The patient's history of transvenous lead dislodgment, involving a 4 French SelectSecure Medtronic 3830 lead, aligns with known challenges in CHD patients. Ng et al. reported late dislodgment of SelectSecure 3830 leads in adolescents with congenital heart block, attributing failures to inadequate atrial loops, fibrous tissue fixation, and somatic growth [1]. In this case, the patient's growth from age 20 to 28, combined with an active lifestyle, likely contributed to lead dislodgment, with Twiddler's syndrome or mechanical stress potential cofactors [1, 2].

The bidirectional Glenn shunt, a palliative procedure redirecting superior vena cava flow to the pulmonary arteries, poses unique challenges for transvenous pacing due to altered venous anatomy and scarring from prior surgeries [3]. Traditional transvenous leads, such as the SelectSecure 3830, are prone to dislodgment or fracture in growing patients, as evidenced by the patient's history and Ng et al.'s case series [1]. Leadless pacemakers eliminate these risks by residing entirely within the RV, offering a durable solution for CHD patients with pacing needs. Beccarino et al. demonstrated midterm success of leadless pacemakers in pacemaker‐dependent patients post‐transvenous lead extraction, reporting stable thresholds and low complication rates [4]. Reynolds et al. further validated the safety and efficacy of leadless pacing systems, with 99.7% successful implantation rates in diverse populations [5].

The procedural innovation of accessing the RV via the Glenn shunt required overcoming significant scarring, highlighting the technical expertise needed in CHD interventions. The use of ultrasound‐guided vascular access and Proglide sutures minimized complications, while angiography and fluoroscopy ensured precise device placement. This approach expands the therapeutic options for patients with single‐ventricle physiology or complex venous anatomy, where epicardial pacing carries higher risks of lead failure and surgical morbidity [6]. The case also underscores the diagnostic value of multimodal imaging, with chest X‐rays, angiography, and echocardiography providing complementary insights into device positioning and cardiac function.

## Author Contributions

R.D. conceptualised the case report, prepared the manuscript, and conducted the literature review. H.C.T. analysed the angiographic images and contributed to manuscript drafting. C.M. aided with additional literature review and manuscript preparation. K.W., as the congenital cardiologist, provided expert clinical input, supervised the procedure, and reviewed the manuscript for accuracy.

## Disclosure

The authors have nothing to report.

## Consent

Informed consent was obtained from the patient for the publication of this case report and accompanying images, in accordance with the ethical standards of the institutional and national responsible committee on human experimentation and the Helsinki Declaration of 1975, as revised in 2013.

## Conflicts of Interest

The authors declare no conflicts of interest.

## Human and Animal Rights

This case report adheres to the ethical standards of the institutional and national responsible committee on human experimentation and complies with the Helsinki Declaration of 1975, as revised in 2013.

## Data Availability

The data that support the findings of this study are openly available in PubMed at https://pubmed.ncbi.nlm.nih.gov/.
